# Characterization of the salivary microbiome in healthy individuals under fatigue status

**DOI:** 10.3389/fcimb.2025.1506723

**Published:** 2025-05-29

**Authors:** Xianhui Peng, Na Han, Yanan Gong, Lihua He, Yanli Xu, Di Xiao, Tingting Zhang, Yujun Qiang, Xiuwen Li, Wen Zhang, Jianzhong Zhang

**Affiliations:** ^1^ National Key Laboratory of Intelligent Tracking and Forecasting for Infectious Diseases, National Institute for Communicable Disease Control and Prevention, Chinese Center for Disease Control and Prevention, Beijing, China; ^2^ Hebei University of Engineering, Affiliated Hospital, College of Medicine, Handan, Hebei, China

**Keywords:** fatigue, healthy individuals, salivary microbiome, 16S rRNA sequencing, biomarkers

## Abstract

**Background & Aims:**

Limited understanding exists regarding the characteristics and biological significance of the salivary microbiome in healthy individuals experiencing physiological fatigue. This study aimed to delineate the structural and functional alterations in the salivary microbiome of healthy individuals undergoing physiological fatigue compared to energetic controls, and to explore its potential as a biomarker for fatigue status.

**Methods:**

A cohort of 7 healthy individuals experiencing acute physiological fatigue (induced by prolonged study and confirmed via electroencephalography; Fatigue group, FTG) and 63 energetic healthy controls (Energetic group, ENG) were enrolled. Saliva samples were collected, from which microbial DNA was extracted. The V3–V4 hypervariable region of the 16S rRNA gene was subsequently sequenced using high-throughput technology. Bioinformatics analyses encompassed assessment of alpha and beta diversity, identification of differential taxa using Linear discriminant analysis Effect Size (LEfSe) with multi-method cross-validation, construction of microbial co-occurrence networks, and screening of fatigue-associated biomarker genera via the Boruta-SHAP algorithm. Microbial community phenotypes and potential functional pathways were predicted using BugBase and PICRUSt2, respectively.

**Results:**

The FTG group exhibited significantly diminished alpha diversity (Simpson index, p=0.01071) relative to the ENG group. Beta diversity analysis demonstrated significant dissimilarities in microbial community structure between the groups (p<0.05). Taxonomic profiling revealed a significant enrichment in the relative abundance of potential periodontopathogenic genera, including *Streptococcus* and *Filifactor*, within the FTG group, concomitantly with a significant depletion of health-associated genera such as *Rothia* and *Neisseria*. A predictive model constructed using the Boruta-SHAP algorithm, based on 15 key genera, effectively discriminated between fatigue and non-fatigue states, achieving an area under the receiver operating characteristic curve (AUC) of 0.948. Phenotypic predictions indicated a significant increase in the proportion of bacteria harboring Mobile Genetic Elements (MGEs) (p=0.048), alongside significant reductions in the proportion of aerobic bacteria (p=0.006) and biofilm-forming capacity (p=0.002) in the FTG group. Functional pathway analysis (PICRUSt2) revealed an enrichment of pathways such as "Neuroactive ligand-receptor interaction" in the FTG group, whereas pathways pertinent to energy metabolism (e.g., Citrate cycle (TCA cycle), Oxidative phosphorylation) and amino acid metabolism (e.g., Phenylalanine metabolism, Histidine metabolism) were significantly enriched in the ENG group.

**Conclusion:**

This study provides novel evidence that physiological fatigue induces significant structural and functional alterations in the salivary microbiome of healthy individuals. These perturbations include diminished microbial diversity, disrupted community architecture, enrichment of potential opportunistic pathogens, and marked shifts in key metabolic pathways, particularly those governing neuroactivity and energy metabolism. These findings suggest that the salivary microbiome may be implicated in the physiological regulation of fatigue, potentially via an "oral-microbiome-brain axis," and underscore its potential as a source of non-invasive biomarkers for assessing fatigue status. Further mechanistic investigations are warranted to elucidate these interactions.

## 1 Introduction

Fatigue is defined as a self-reported functional impairment symptom characterized by limitations in physical and cognitive functions, and it typically involves complex mechanisms such as immune dysfunction, metabolic disorders, and regulation of the microbiota-gut-brain axis (Raizen et al., 2023). Fatigue can be classified as physiological and pathological fatigue. Physiological fatigue, which develops from physical activity, mental exertion, sleep deprivation, or infections, is relieved by rest and/or sleep. Pathological fatigue, resulting from conditions such as myalgic encephalomyelitis/chronic fatigue syndrome (ME/CFS), rheumatic diseases, multiple sclerosis, Parkinson’s disease, and long COVID postacute sequelae of SARS-CoV-2 infection (PASC), is only partially relieved by rest (Raizen et al., 2023). Recently, a systematic review and meta-analysis of the global prevalence of fatigue reported an average prevalence of 7.7% for chronic fatigue (pathological fatigue) and 24.2% for generalized fatigue (physiological fatigue) (Yoon et al., 2023). Fatigue has a substantial economic impact on society. For instance, it is estimated to cost employers over $136 billion annually in the United States due to the loss of productivity (Ricci et al., 2007). However, this estimate does not account for additional losses due to accidents related to fatigued driving (Zhang et al., 2022) and negative health outcomes associated with fatigue (Knoop et al., 2021). The impact of fatigue on healthcare is severely underestimated. Despite the high economic and social costs of fatigue, the mechanisms and biomarkers of fatigue under different health conditions remain unclear.

Over the past two decades, remarkable advances have been made in microbiome research (Fremont et al., 2013; Nagy-Szakal et al., 2017; Guo et al., 2023; Xiong et al., 2023). Several studies have investigated the relationship between the gut microbiome and fatigue (Fremont et al., 2013; Nagy-Szakal et al., 2017; Guo et al., 2023; Xiong et al., 2023). Previous research indicates that the gut microbiome composition in ME/CFS patients is altered, with a reduction in biodiversity; however, the precise relationship between bacterial composition and ME/CFS pathogenesis remains unclear. Dysbiosis may affect ME/CFS through the microbiota-gut-brain axis in several potential ways. These include (1) inflammation and immune activation: Dysbiosis can lead to increased intestinal permeability, commonly known as “leaky gut,” which allows bacteria or bacterial metabolites from the gut to enter the bloodstream. This can trigger immune responses and systemic inflammation, thereby affecting the brain and contributing to ME/CFS symptoms (Clapp et al., 2017); (2) neurotransmitter signaling: The gut microbiome plays a role in producing and regulating neurotransmitters. Dysbiosis can disrupt the production and balance of neurotransmitters such as serotonin (5-HT) and γ-aminobutyric acid (GABA), which are crucial for mood, cognition, and other brain functions. Alterations in neurotransmitter production and signaling may contribute to fatigue symptoms in ME/CFS patients (Loebel et al., 2016); (3) metabolite production: The gut microbiome produces various metabolites, including short-chain fatty acids (SCFAs), which can influence brain function and behavior. Dysbiosis may alter the production and availability of these metabolites, thereby potentially affecting gut-brain communication and leading to ME/CFS symptoms; and (4) activation of the immune-brain axis: Dysbiosis can activate the immune system, leading to the release of proinflammatory cytokines and other immune molecules. These immune molecules can communicate with the brain through pathways such as the vagus nerve and immune cell transport, potentially affecting brain function and contributing to ME/CFS symptoms (Holzer et al., 2017; Arzani et al., 2020).

While research on the microbiota-gut-brain axis has substantially enhanced our understanding of the interactions between the microbiota and fatigue, it has predominantly focused on the lower gastrointestinal tract, often overlooking another crucial environment: the oral microbiome. The oral cavity is the entry point for all substances (microorganisms and other substances) into the body and serves as the starting point of the digestive system. Similar to research on the gut microbiome, oral microbiome research is shifting toward a comprehensive understanding of its functions and interactions with the body (Baker et al., 2024). Recent findings indicate that the oral microbiome is not only a marker of oral health issues, such as dental caries and periodontal disease, but also a key player in systemic conditions, including obesity, diabetes, and neurological and psychiatric disorders (Wu et al., 2018; Cunha et al., 2019; Lin et al., 2019; Xue et al., 2020; Yang et al., 2021; Ahrens et al., 2022). Indeed, similar to the gut microbiome, the oral microbiome may also engage in complex bidirectional interactions between the brain and the central nervous system (CNS). The cascading effects of the oral microbiota and metabolites escaping into the brain can directly lead to the development various diseases. For instance, in mice, the oral pathogen *Streptococcus mutans* can enter the bloodstream from the oral cavity and induce cerebral hemorrhage by disrupting the blood-brain barrier through its collagen-binding activity (Watanabe et al., 2016). Similarly, *Porphyromonas gingivalis*, a bacterial species present in many individuals with poor oral health, may play a pivotal role in the development and progression of periodontal disease. Notably, *P. gingivalis* can enter the bloodstream, colonize the brain, and release neurotoxic proteases known as gingipains, which are implicated in Alzheimer’s disease progression (Lassalle et al., 2018). Recent studies have revealed how the oral microbiome negatively affects neurological processes and influences cognition and behavior. The analysis of the oral microbiome metabolic pathways in smokers showed enrichment of the neurotransmitter-related pathways. These pathways include tyrosine metabolism and the production of glutamine-glutamate and glutamatergic synapses. Smoking stimulates neurotransmitter production through the glutamine and glutamate pathways, thereby influencing reward circuitry in the brain. Thus, the oral microbiome can directly affect the reward pathways associated with smoking behavior and dependence, altering the typical interactions between the oral microbiome and the brain’s functional connectivity (Lin et al., 2019).

In light of this research background, to determine the relationship between the oral microbiome and fatigue, we performed 16S rRNA high-throughput sequencing to analyze the oral microbiome composition in a cohort of fatigued healthy individuals. We further predicted the phenotypes and metabolic pathways of the oral microbiome in these fatigued subjects. By evaluating the effect of fatigue on the oral microbiome, we aimed to infer the potential implications of these changes for oral health, the CNS, and overall systemic health.

## 2 Materials and methods

### 2.1 Study design, participants, and assessment procedures

This study employed a prospective observational design, with the detailed protocol previously described (Xu et al., 2018; Xu et al., 2020). Seventy healthy university students, aged 18–50 years, were recruited. Baseline assessments confirmed all participants had sufficient sleep, exhibited normal awake electroencephalogram (EEG) patterns (absence of fatigue characteristics), and did not meet criteria for ME/CFS. Stringent inclusion and exclusion criteria (adapted from Breithaupt-Groegler et al (Breithaupt-Groegler et al., 2017), covering chronic fatigue history, recent infections, specific symptoms, medication use, smoking, and oral health) were rigorously applied. Ethical approval was obtained from the Ethics Committee of the Affiliated Hospital of Hebei University of Engineering (Handan, China; March 12, 2014; Clinical Trial Registration: ChiCTR-DCD-14005746), and written informed consent was secured from all participants. Subsequently, all 70 eligible participants underwent a standardized physiological fatigue induction protocol, involving continuous high-intensity cognitive tasks (“continuous study work”) in a quiet setting for at least 18 hours (actual range: 18–24 hours), with minimal necessary breaks.

Immediately following the cognitive tasks, comprehensive subjective and objective fatigue assessments were conducted on all participants. Subjective fatigue was evaluated using a revised Piper Fatigue Scale (PFS; based on Piper et al (Piper et al., 1998)), with item scores from 0-10; the overall average score categorized fatigue as mild (1-3.3), moderate (3.4-6.7), or severe (6.8-10), serving as a preliminary reference for grouping. Objective fatigue state was determined via EEG monitoring (SOLAR-RTA/BFM system), based on characteristic waveform changes compared to baseline (significant increase in slow-wave and/or decrease in fast-wave activity). Upon completion of these assessments, saliva samples were immediately collected from all 70 participants for subsequent microbiome analysis.

The final group assignment for the comparative analyses herein was strictly determined based on the objective EEG assessment results, although PFS scores provided valuable subjective context. The Fatigue Group (FTG; n=7) comprised participants whose post-protocol EEG clearly met the predefined objective fatigue criteria (typically corresponding with higher PFS scores, e.g., >6.7). Conversely, the Energetic Group (ENG; n=63) consisted of participants who, despite completing the same protocol, did not meet the objective EEG fatigue criteria (typically corresponding with lower PFS scores, e.g., ≤3.3).

### 2.2 Saliva sample collection

Saliva samples were collected from the subjects after 18 h of continuous study work. The subjects rinsed their mouths three times with sterile saline. Prior to sample collection, all subjects rinsed their mouth three times (1 min each time) with 30 mL of distilled water to remove food debris. After rinsing, each subject sat straight in a seat for 5 min, with their head tilted slightly forward and their eyes open. The subjects then chewed to stimulate salivary secretion. Once a sufficient amount of saliva had accumulated in the lower jaw, they placed their tongue against the palate and opened their mouth to allow the saliva to flow naturally into a 2 mL sterile centrifuge tube. Subsequently, the saliva samples from each participant were collected and immediately transported to the National Key Laboratory of Institute of Infectious Diseases Prevention and Control of Chinese Center for Disease Control and Prevention by a cold-chain shipping company and stored at -80°C.

### 2.3 DNA extraction and 16S rRNA gene sequencing

An aliquot of 500 μL saliva was centrifuged at 13,200 rpm for 10 min. The supernatant was discarded, and the precipitate was retained. Total saliva microbial DNA was extracted using the QIAamp^®^ DNA Mini Kit (Qiagen, Germany) following the manufacturer’s instructions. The quantity and quality of extracted DNA were assessed using a NanoDrop 2000 spectrophotometer and agarose gel electrophoresis, respectively. The extracted DNA was used as a template for the PCR amplification of bacterial 16S rRNA genes of the V3–V4 region with specific primers containing barcodes. The primer sequences were 341F (5′-CCTAYGGGRBGCASCAG-3′) and 806R (5′-GGACTACNNGGGGTATCTAAT-3′) (Youngseob et al., 2005). The amplicons were purified, quantified, and prepared for library construction, with all amplicons mixed in equal amounts. Library quality was assessed on the Qubit^®^ 2.0 fluorometer (Thermo Scientific) and the Agilent Bioanalyzer 2100 system. The library was sequenced on an Illumina HiSeq 2000 platform (250 bp paired-end reads) at Novogene Bioinformatics Technology. (Beijing, China).

### 2.4 Sequencing data processing and bioinformatics analysis

Raw sequencing reads were processed using the USEARCH pipeline (v11.0.667) for amplicon sequence analysis (Edgar, 2010). Key processing steps included demultiplexing reads based on barcodes and performing quality filtering. Subsequently, the UNOISE3 algorithm was employed for sequence denoising, merging of paired-end reads and generation of non-chimeric Amplicon Sequence Variants (ASVs) (Edgar, 2016a). The UCHIME process inherently includes chimera detection and removal (Edgar, 2016b). Taxonomic assignment of the resulting representative ASV sequences was performed using the RDP Classifier (v2.13) against a relevant reference database (Cole et al., 2014).

Alpha diversity indices (Shannon, Simpson, Chao1, and ACE) were calculated to assess within-sample microbial diversity after rarefying each sample to a depth of 10,000 reads. To evaluate beta diversity (between-sample community structure differences), the robust Aitchison distance, which is suitable for compositional microbiome data, was calculated using the vegan package (v2.6-6.1) in R (v4.2.3) (Clarke, 1993) (Oksanen, 2024). Non-metric Multidimensional Scaling (NMDS) ordination based on the Aitchison distance matrix was used to visualize community structures, and the Wilcoxon rank-sum test was applied to assess significant differences between groups in the NMDS ordination space. Additionally, Permutational Multivariate Analysis of Variance (PERMANOVA) and Multi-Response Permutation Procedures (MRPP) were conducted to formally test for significant differences in overall community composition between groups. For PERMANOVA and MRPP analysis, the function of the vegan package for R was used. Linear Discriminant Analysis Effect Size (LEfSe) (v1.1.2) was utilized to identify statistically significant differentially abundant taxa between groups, applying a Linear Discriminant Analysis (LDA) score threshold of > 3.0 (Segata et al., 2011). To ensure the robustness of the identified differential taxa, the results were cross-validated using multiple alternative methods, including ALDEx2 (Gloor, 2015), ANCOM-II (Mandal et al., 2015), MaAsLin3 (Nickols et al., 2024), PROC-GLM (Sunwoo et al., 2020) and ZicoSeq (Yang and Chen, 2023).

Microbial co-occurrence networks were constructed using the R package ggClusterNet (v0.1.0). Network edges were determined based on SparCC correlations (|r| > 0.3, p < 0.05) between taxa (Wen et al., 2022). The functional potential of the microbial communities was predicted by inferring KEGG Orthology (KO) profiles from ASV data using PICRUSt2 (v1.7.2) (Douglas et al., 2020). Differential abundance analysis of predicted functional pathways between groups was performed using the LinDA method implemented within the ggpicrust2 package (v1.7.2), which also facilitated visualization (Yang et al., 2023). Furthermore, key phenotypic traits of the microbial communities, such as Gram staining properties, oxygen tolerance, and biofilm formation capacity, were inferred using BugBase (https://github.com/knights-lab/BugBase) (Ward et al., 2017).

To identify potential taxonomic biomarkers associated with fatigue status, a feature selection approach using the Boruta algorithm (implemented with LightGBM) was employed (Lundberg and Lee, 2017). SHAP (SHapley Additive exPlanations) analysis was subsequently applied to interpret the contribution of the selected features to the model, thereby enhancing model interpretability (Kursa and Rudnicki, 2010).

### 2.5 Statistical analysis

Baseline demographic characteristics were compared between the Fatigue Group (FTG) and the Energetic Group (ENG). Specifically, continuous variables (e.g., age) were compared between groups using PROC GLM (implemented via the sasLM package v0.10.3 in R v4.2.3). *Post hoc* analysis using Tukey’s Honestly Significant Difference (HSD) test was performed if required for multiple comparisons. Categorical variables (e.g., gender distribution) were compared using the Wilcoxon rank-sum test (or Chi-squared test, as appropriate for the data).

Statistical methods for group comparisons related to bioinformatics analyses (e.g., comparisons of alpha diversity indices, PERMANOVA/MRPP tests for beta diversity, differential abundance analysis of taxa and functions) are detailed within their respective descriptions in Section 1.4.

All statistical tests were two-sided where applicable. A p-value < 0.05 was considered statistically significant. Significance levels in the results are denoted as *p < 0.05, **p < 0.01, and ***p < 0.001.

## 3 Results

### 3.1 Overview of the study cohort and sequencing data

**Figure 1:**
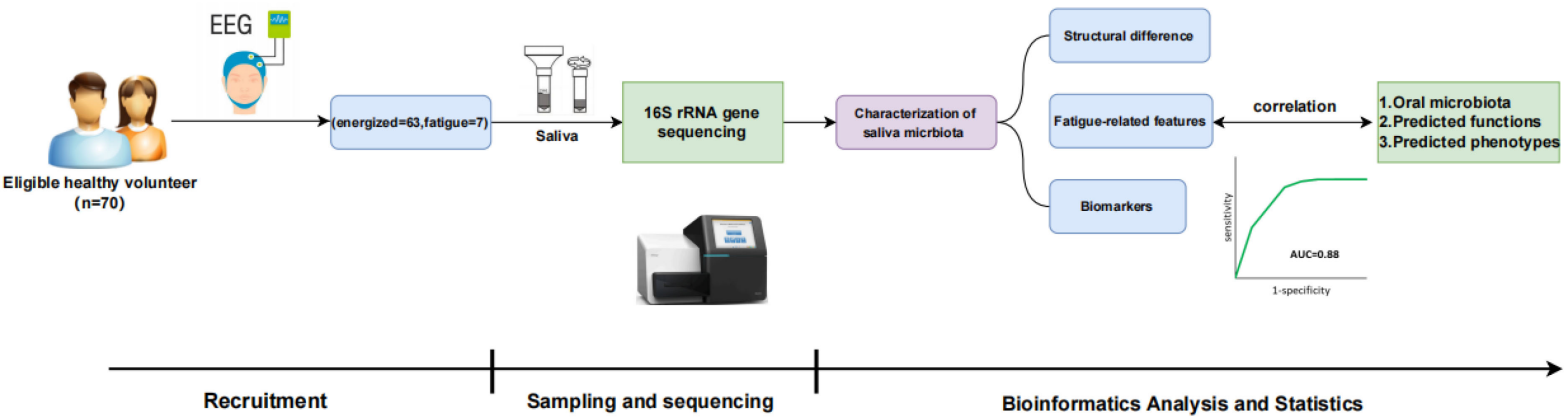
Schematic overview of the study workflow. Healthy volunteers (n=70) were recruited and assessed (including EEG), leading to categorization (energized=63, fatigue=7). Saliva samples underwent 16S rRNA gene sequencing. Bioinformatics analysis characterized the saliva microbiota to identify structural differences, fatigue-related features, and biomarkers, which were correlated with microbiota composition, predicted functions, and phenotypes. Key phases included Recruitment, Sampling and Sequencing, and Bioinformatics Analysis and Statistics.

A total of 70 healthy individuals aged 18–50 years were enrolled according to strict inclusion and exclusion criteria (**Figure 1**), including 7 individuals experiencing fatigue (FTG) and 63 individuals in an energized state (ENG). No significant differences in gender or age were observed between the two groups (**Supplementary Table S1**). Saliva samples were systematically collected from all participants and subjected to 16S rRNA amplicon sequencing on the Illumina HiSeq 2000 platform. Following rigorous data preprocessing and quality control procedures—including splicing, filtering, and chimera removal—4,164,405 valid sequences were obtained in total, with each sample yielding an average of 59,492 ± 5,101 sequences. The average read length of the valid sequences was 425 base pairs. Subsequent taxonomic assignment revealed 1,204 ASVs, providing a comprehensive overview of the microbial profiles in these samples.

### 3.2 Intra-variations in salivary microbial diversity between the FTG and ENG groups

**Figure 2:**
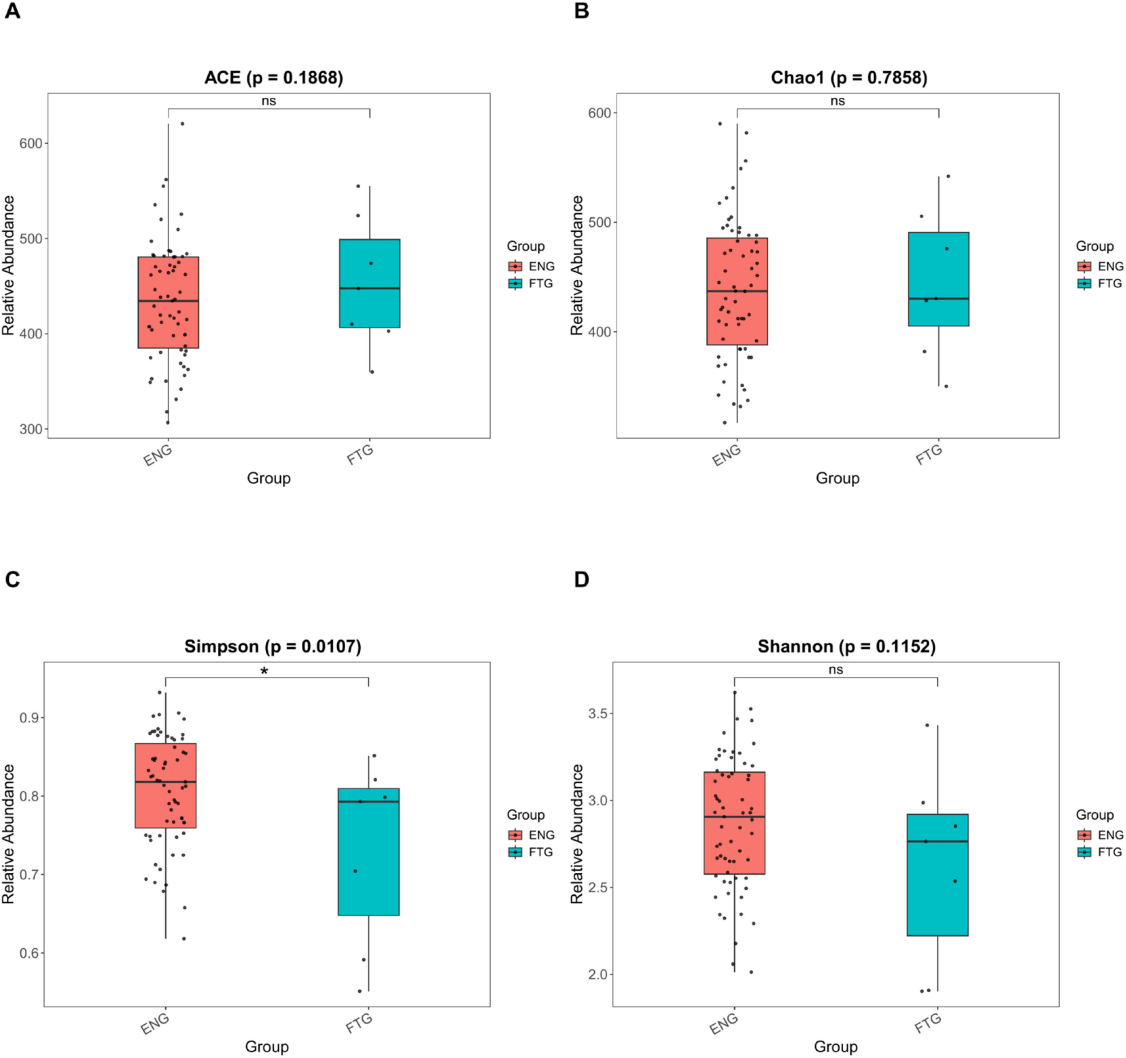
Comparison of α-diversity indices of the oral microbiota between the FTG and ENG groups. **(A)** ACE index. **(B)** Chao1 index. **(C)** Shannon index. **(D)** Simpson index. Statistical comparisons were performed using the PROC GLM test, with significance levels denoted as follows: P < 0.05 (*) and P > 0.05 (ns).

To validate sequencing depth adequacy for microbial diversity assessment, rarefaction curves were initially constructed to confirm data saturation (**Supplementary Figure S1**). Comparative analysis of α-diversity indices revealed a significant reduction in overall microbial diversity within the FTG group compared to controls (**Supplementary Table S2**). Regarding indices predominantly reflecting species richness, elevated values were observed in the FTG group for both ACE (439.22 ± 59.84, p = 0.1868, **Figure 2A**) and Chao1 (438.67 ± 61.66, p = 0.7858, **Figure 2B**) estimators. Interestingly, while the Simpson index (0.80 ± 0.08, p = 0.01071, **Figure 2C**) and Shannon index (2.85 ± 0.40, p = 0.1152, **Figure 2D**) integrate both species richness and evenness, only the Simpson index demonstrated statistically significant intergroup differences. This discrepancy suggests potential dominance of specific microbial taxa in community structure of the FTG group.

### 3.3 Inter-variations in salivary microbial diversity between the FTG and ENG groups

**Figure 3:**
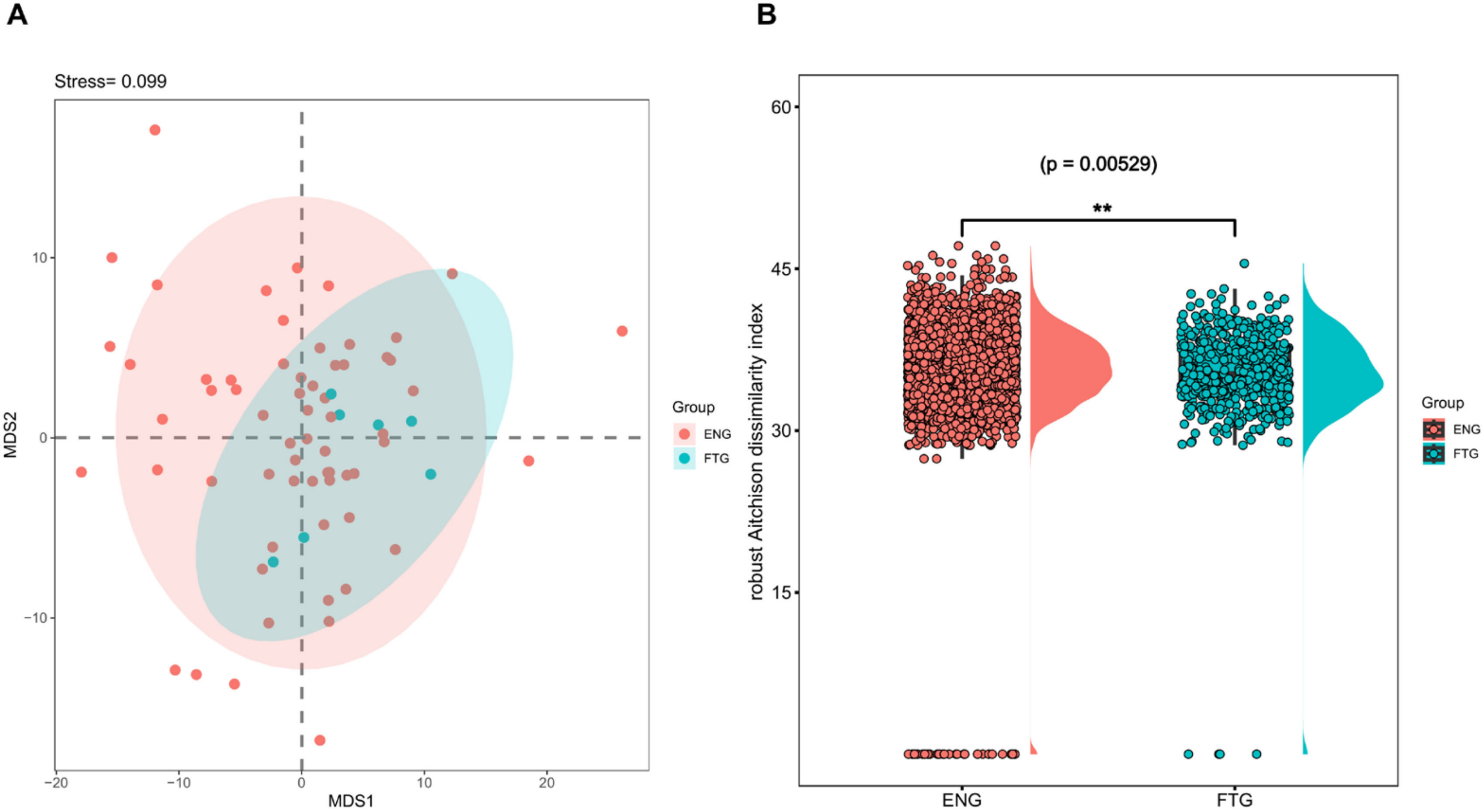
Comparison of β-diversity indices of the oral microbiota between the FTG and ENG groups. **(A)** Non-metric multidimensional scaling (NMDS) ordination derived from robust Aitchison dissimilarity distances for oral microbiota community (stress value = 0.099, k = 2). Colored ellipses indicate 95% confidence intervals for each group. **(B)** The boxplot illustrates the distribution of robust Aitchison distances for pairwise comparisons between samples from the FTG and ENG groups. Individual data points represent specific pairwise comparisons, with asterisks (**) denoting statistically significant differences between groups as determined by Wilcoxon rank-sum test (p < 0.01).

To evaluate the similarity of salivary microbial communities, β-diversity analysis at the amplicon sequence variant (ASV) level was performed on 16S rRNA amplicon sequencing data using the vegan package (v2.6.4). A Robust Aitchison distance matrix, optimized for compositional data analysis, was constructed to characterize microbial community structures. Non-metric multidimensional scaling (NMDS) was employed to visualize inter-group differences between FTG and ENG cohorts (**Figure 3A**). While NMDS plot demonstrated partial overlap between groups, suggesting subtle overall differences in salivary microbiota, statistically significant distinctions were confirmed through Wilcoxon rank-sum test (**Figure 3B**, p=5.29E-3), ANOSIM (permutations=999, p=0.029), and MRPP (permutations=9999, p=0.0419).

### 3.4 Taxonomic differences in the salivary microbiome of the FTG and ENG groups

**Figure 4:**
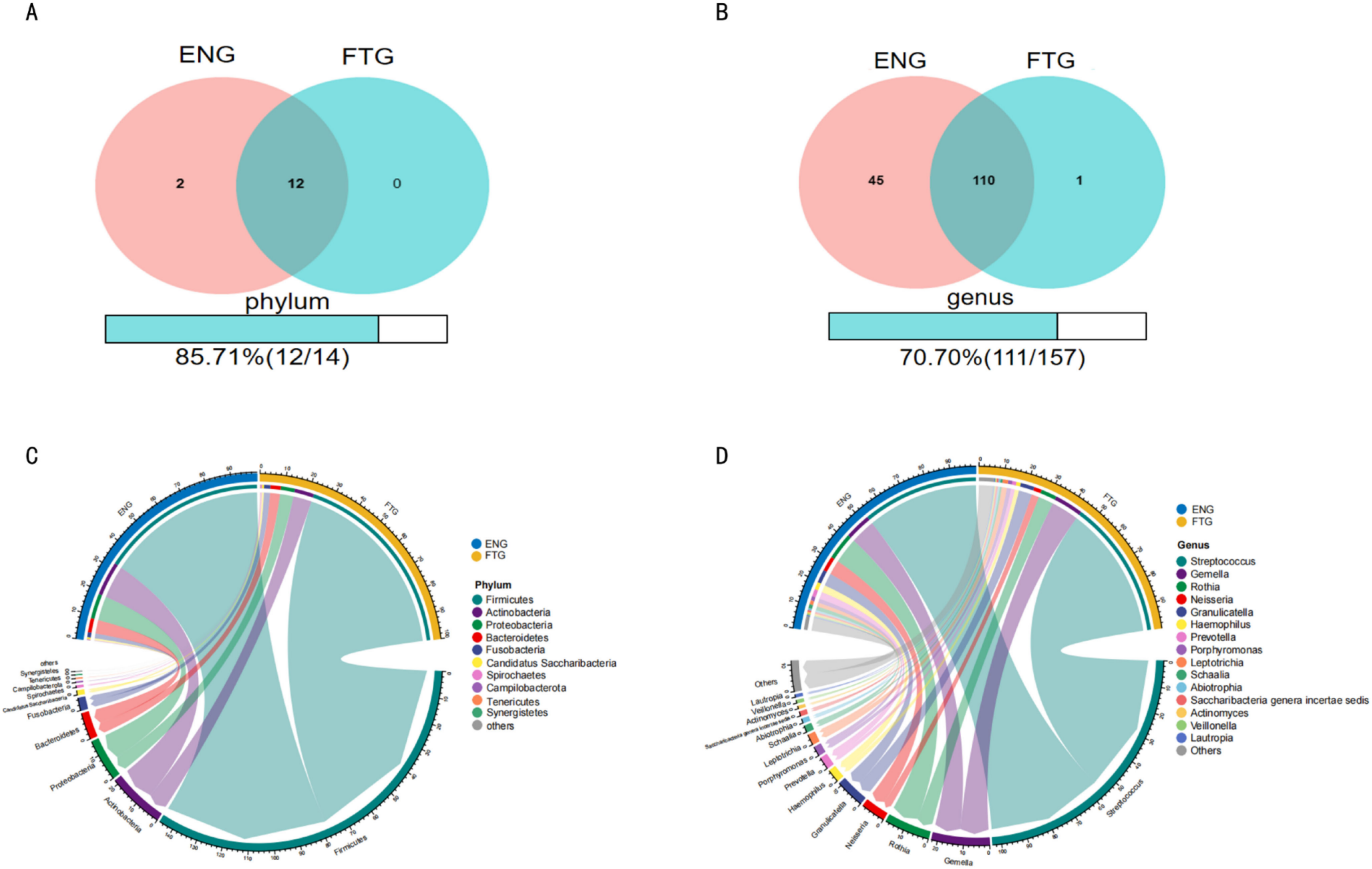
Comparison of salivary microbial community composition between ENG and FTG groups. **(A, B)** Venn diagrams assessing the number of shared and unique taxa at the phylum **(A)** and genus **(B)** levels. Numbers are counts; percentages indicate shared proportion. **(C, D)** Circos plots illustrating relative abundance and inter-group associations of major taxa at the top 10 phylum **(C)** and top 15 genus **(D)** levels in ENG (left) and FTG (right) groups. Outer arc length corresponds to relative abundance; inner ribbon width reflects association strength.

To elucidate compositional differences in the salivary microbiota between energized (ENG) and fatigued (FTG) individuals at the phylum and genus levels, a systematic comparative analysis was conducted. First, Venn diagrams were employed to quantitatively assess shared and unique microbial taxonomic units between the groups (**Figures 4A, B**). Phylum-level analysis (**Figure 4A**) identified 14 phyla in total, with 12 shared by both groups. Two phyla (Chloroflexi and Elusimicrobia) were unique to the ENG group, resulting in a high shared proportion (85.71%) and indicating substantial similarity in core phylum composition. Genus-level analysis (**Figure 4B**), however, revealed that among the 157 identified genera, only 111 were shared. The ENG group harbored significantly more unique genera (n=45) compared to the FTG group (n=1), with the shared proportion decreasing to 70.70%. This highlights increased inter-group divergence and greater microbial uniqueness within the ENG cohort at the genus level.

Second, Circos plots were utilized to visualize the relative abundance and inter-group associations of major taxa (**Figures 4C, D**). The phylum-level Circos plot (**Figure 4C**) displayed the top 10 most abundant phyla (cumulatively >99.9% relative abundance), confirming Firmicutes, Proteobacteria, Bacteroidetes, and Actinobacteria as the primary dominant phyla in both groups. The genus-level Circos plot (**Figure 4D**) focused on the top 15 most abundant genera (each >1% relative abundance, collectively >60% total relative abundance), clearly illustrating significant inter-group differentiation: *Streptococcus* exhibited markedly higher relative abundance in the FTG group, whereas *Rothia* and *Neisseria* were notably enriched in the ENG group. Furthermore, abundance differences for other major genera, including *Gemella*, *Granulicatella*, and *Prevotella*, were also depicted.

### 3.5 Oral microbiome network in healthy individuals in fatigue state

**Figure 5:**
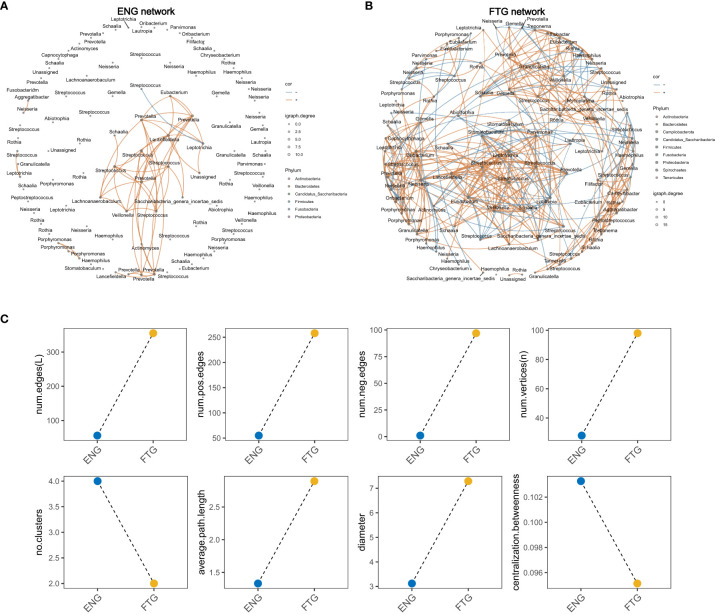
Salivary microbial network of the ENG and FTG groups. **(A)** Visualization of the salivary microbial network in the ENG group. **(B)** Visualization of the salivary microbial network in the FTG group. **(C)** Changes in network topology, including number of clusters (No. Clusters), number of edges (Num. Edges), average path length, and diameter between the two groups.

To elucidate the interactions within the oral microbiome, we identified interactive networks within the groups (**Figures 5A, B**) and delineated differences in microbiome interactions between the FTG and ENG groups (**Figure 5C**). We found interactive networks within the oral microbiome of the ENG group (**Figure 4A**). This was confirmed by multiple network topological indices, whose values were greater than zero in the saliva samples of the ENG group, including the number of clusters (No. Clusters), number of edges (Num. Edges), number of positive edges (Num. Pos. Edges), number of negative edges (Num. Neg. Edges), number of vertices, diameter, average path length, and centralization betweenness (**Supplementary Table S3**); this finding indicated the existence of a complex network of microbiota in the ENG group (**Figure 5A**). The interacting microbiota within the networks of the ENG group was predominantly distributed across 6 phyla and 13 genera. These genera, ranked in descending order of interaction frequency, were as follows: *Prevotella* (40/112, 35.71%), *Streptococcus* (26/112, 23.21%), *Lancefieldella* (11/112, 9.82%), *Leptotrichia* (7/112, 6.25%), *Porphyromonas* (6/112, 5.36%), *Lachnoanaerobaculum* (5/112, 4.46%), *Veillonella* (5/112, 4.46%), *Eubacterium* (4/112, 3.57%), *Saccharibacteria_genera_incertae_sedis* (2/112, 1.79%), Unassigned (2/112, 1.79%), *Aggregatibacter* (1/112, 0.89%), *Granulicatella* (1/112, 0.89%), *Neisseria* (1/112, 0.89%), and *Schaalia* (1/112, 0.89%).

Concurrently, we observed an increase in the network topological complexity in the FTG group, as indicated by metrics such as Num. Edges, Num. Pos. Edges, Num. Neg. Edges, number of vertices, and diameter (**Figures 5B, C**; **Supplementary Table S3**). The dashed line plot (**Figure 5C**) indicates a significant increase in both the number of edges and vertices in the network topology of the FTG group. The numbers of both positively and negatively correlated edges were significantly higher in the FTG group than in the ENG group. However, the FTG group showed a decrease in network topological indices such as connectance (edge density) and mean clustering coefficient (average CC), which suggested a reduction in network cohesiveness in this group.

Specifically, the FTG group network included interacting species from 11 phyla and 33 genera. The top 15 species based on interaction frequency were *Streptococcus* (123/710, 17.32%), *Neisseria* (54/710, 7.61%), *Rothia* (47/710, 6.62%), *Schaalia* (46/710, 6.48%), *Prevotella* (38/710, 5.35%), *Veillonella* (33/710, 4.65%), *Leptotrichia* (32/710, 4.51%), *Porphyromonas* (30/710, 4.23%), *Gemella* (28/710, 3.94%), *Eubacterium* (26/710, 3.66%), *Oribacterium* (26/710, 3.66%), *Stomatobaculum* (26/710, 3.66%), *Lautropia* (20/710, 2.82%), *Granulicatella* (19/710, 2.68%), and *Haemophilus* (18/710, 2.56%). These findings revealed that under fatigue conditions, the number of interacting species and the frequency of their interactions in the FTG group network significantly increased, along with a notable increase in both cooperative and competitive interactions among the species. However, despite the increased complexity of the network, its overall density decreased.

### 3.6 Identification and validation of salivary microbiota biomarkers for fatigue status

**Figure 6:**
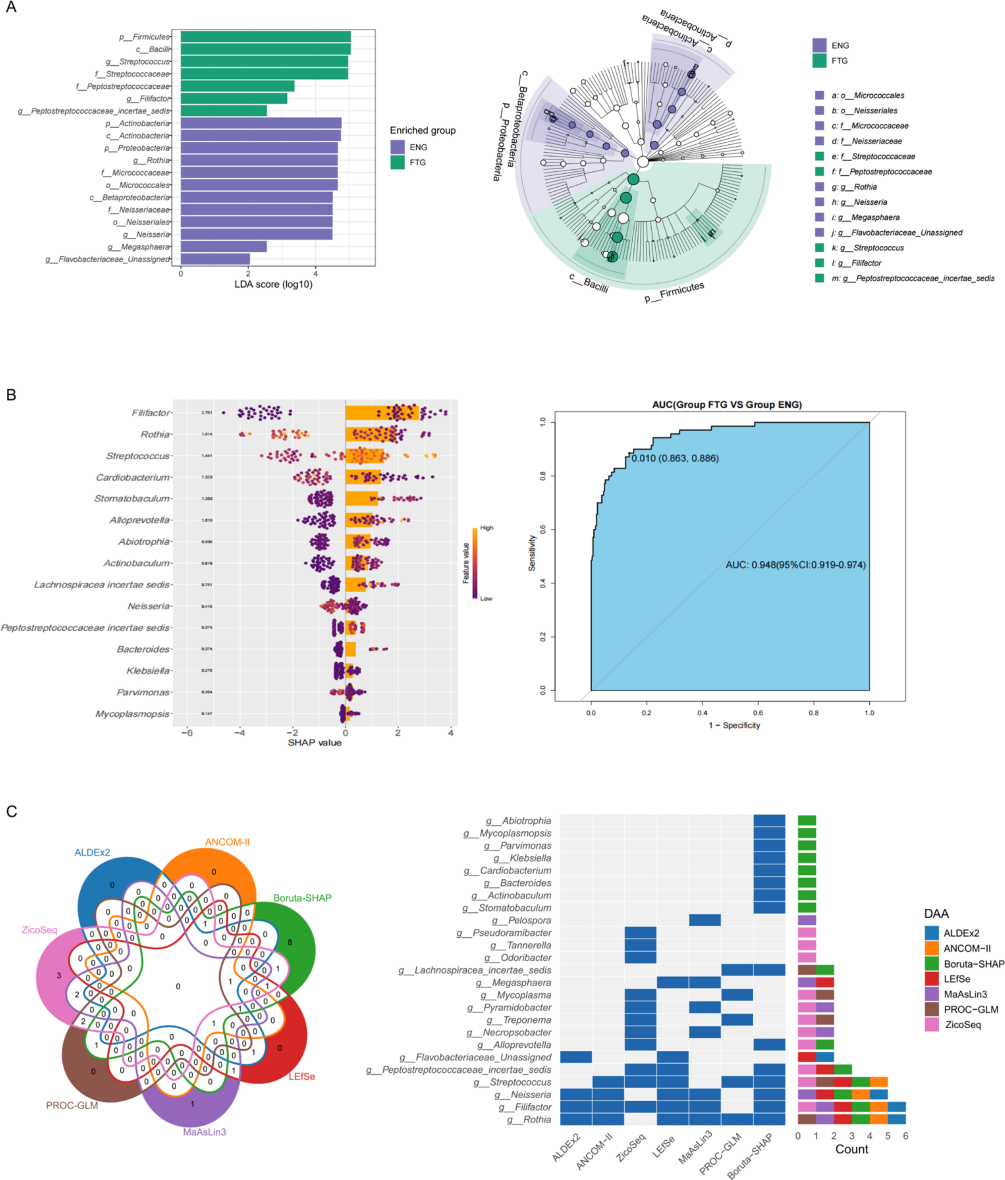
Biomarker taxa of the salivary microbiota in the FTG and ENG groups. **(A)** Intergroup microbial community markers of the oral microbiota in the ENG and FTG groups based on the LEfSe analysis. **(B)** SHAP summary plots according to the Boruta-SHAP algorithm. Bar plot showing global SHAP values for feature importance, and beeswarm plot for the local SHAP values, showing the contribution of each genus to the fatigue predictions of the model. Features in both bar plot and beeswarm plot were ranked by mean absolute SHAP value, hence their rankings are identical. In the SHAP beeswarm plot, each point represents an individual in the training data. The x-axis corresponds to the SHAP value, with vertical jitter indicating a high density of points. The color scale indicates the relative magnitude of each feature with yellow indicating high values of the feature and purple the opposite; ROC curve obtained for the 15 feature genera from the model based on the Boruta-SHAP algorithm. **(C)** Venn diagram and heatmap visualizations comparing genus-level taxa detected by seven differential analysis methods. The Venn diagram illustrates the overlap of genus-level taxa detected by each method. The heatmap displays the distribution patterns of the detected genus-level species across different methods, allowing for a visual comparison of their performance.

To elucidate the distinctive salivary microbiome profiles associated with the fatigue state (FTG), we initially employed Linear Discriminant Analysis Effect Size (LEfSe) to compare microbial taxa with significantly different abundances between the FTG and ENG groups. The resulting cladogram illustrates the differentially abundant taxa hierarchically from phylum to genus level. Nineteen taxa were identified with significant differential abundance (LDA score > 2, P < 0.05) between the groups (**Figure 6A**). Specifically, the FTG group exhibited significant enrichment of Firmicutes(phylum), Bacilli(class),Streptococcaceae(family), Peptostreptococcaceae(family),*Streptococcus* (genus), *Filifactor* (genus),and *Peptostreptococcaceae incertae sedis* (unclassified genus). Conversely, the ENG group demonstrated significantly higher abundances of Actinobacteria (phylum), Actinobacteria (class), Micrococcales (order), Micrococcaceae(family),Proteobacteria(phylum),Betaproteobacteria(class),Neisseriales(order),Neisseriaceae(family), *Rothia* (genus), *Neisseria* (genus), *Megasphaera* (genus),and *Flavobacteriaceae_Unassigned* (unclassified genus).

To investigate the predictive utility of the salivary microbiome in discriminating individual fatigue status, this study constructed a machine learning model based on genus-level taxonomy. The model employed the Boruta algorithm for feature selection and integrated SHAP (SHapley Additive exPlanations) analysis for model interpretation and key feature identification, with its performance and robustness ultimately assessed via cross-validation. The model’s predictive efficacy, evaluated using the Receiver Operating Characteristic (ROC) curve (**Figure 6B**), yielded an Area Under the Curve (AUC) of 0.948 (95% CI: 0.919 - 0.974), demonstrating excellent and statistically significant discriminatory power in effectively differentiating between fatigued and non-fatigued (energized) individuals. Furthermore, assessment of model stability through resampling techniques revealed robust performance across key metrics (**Supplementary Figure S2**), exhibiting high consistency particularly for Specificity (mean ~0.95), Negative Predictive Value (mean ~0.94), and AUC (mean ~0.95). Accuracy (mean ~0.90) and Positive Predictive Value (mean ~0.84) also showed good performance. Although Sensitivity (mean ~0.75) and F1 Score (mean ~0.79) were comparatively lower with slightly wider distributions, suggesting potential room for improvement in identifying fatigued (positive) samples, the Matthews Correlation Coefficient (MCC, mean ~0.71), as a balanced metric, nonetheless confirmed the model’s reasonably good overall predictive capability. To gain deeper insights into the model’s decision-making mechanisms, SHAP analysis was utilized to visualize the contributions of the top 15 feature genera (**Figure 6B**). The SHAP summary plot (comprising a beeswarm plot and a bar plot) clearly elucidated: (1) feature importance ranking based on mean absolute SHAP values; (2) the directionality of the effect of feature abundance (color: yellow=high, purple=low) on predictive contribution (sign of SHAP value); and (3) the pattern of the relationship between feature abundance and predictive impact (distribution of points). For instance, *Rothia*, the most important feature, showed that higher abundance was associated with a reduced prediction of fatigue (predominantly negative SHAP values), and its relatively symmetrical SHAP value distribution suggested an approximately linear relationship between its abundance and fatigue status risk. In summary, this study successfully developed and validated a high-performance classifier based on the salivary microbiome, capable of reliably discriminating between different fatigue statuses (AUC=0.948). The model demonstrated robust performance, and SHAP analysis elucidated the specific impact patterns of key microbial taxa (e.g., *Rothia*) and their abundances on predictions, enhancing model interpretability. These findings indicate that the identified key salivary microbial taxa hold potential value as non-invasive biomarkers for clinical fatigue risk assessment.

To robustly validate the bacterial genera significantly associated with fatigue, we employed a cross-validation strategy using seven distinct differential abundance analysis methods: LEfSe, ALDEx2, ANCOM-II, ZicoSeq, MaAsLin3, PROC-GLM, and our previously described fatigue-associated Boruta-SHAP algorithm model. Comparative visualization via Venn diagrams and a heatmap facilitated the assessment of consensus in genus-level taxa identification across these methodologies (**Figure 6C**). The Venn diagram illustrates the overlap of genera detected by each method, highlighting methodological concordance. The heatmap depicts the distribution patterns (e.g., detection status or statistical significance/effect size) of the consensus genera across the analytical approaches, enabling a visual comparison of their performance. Thirteen genera exhibited consistent detection by at least two methods. Notably, *Rothia* and *Filifactor* were concurrently identified by six methodologies, followed by *Neisseria* and *Streptococcus*, detected by five approaches. *Peptostreptococcaceae incertae sedis* demonstrated consensus across three methods. Eight additional genera (*Megasphaera*, *Mycoplasma*, *Pyramidobacter*, *Treponema*, *Necropsobacter*, *Pseudoramibacter*, *Alloprevotella*, and *Flavobacteriaceae_Unassigned*) showed agreement between two independent analytical frameworks. This comprehensive multi-method validation strategy substantially strengthens the reliability of these microbial signatures as fatigue-associated biomarkers.

**Figure 7:**
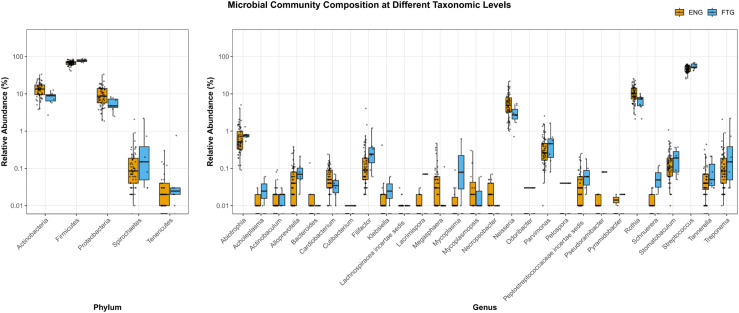
Comparison of salivary microbial community composition between the ENG and FTG groups at the phylum and genus levels. Box plots show the relative abundance (%) distribution (Y-axis, log10 scale) of major taxa at the phylum (left panel) and genus (right panel) levels. Orange boxes represent the ENG group, and blue boxes represent the FTG group. Each box indicates the interquartile range (IQR), the horizontal line within the box represents the median, and the whiskers extend to the furthest data points within 1.5 times the IQR from the box edge. Individual dots represent the actual relative abundance values for each sample corresponding to the respective taxon.

Furthermore, to visually compare the abundance distributions of potential microbial biomarkers within saliva samples from the ENG and FTG groups, boxplots were generated (**Figure 7**). These plots illustrate the relative abundances (Y-axis, log10 scale) of major microbial taxa at the phylum (left panel) and genus (right panel) levels between the two groups. At the phylum level, Firmicutes and Proteobacteria were identified as the most dominant phyla in both groups. Compared to the FTG group, the ENG group showed slightly higher trending relative abundances of Actinobacteria and Proteobacteria, whereas the relative abundance of Firmicutes appeared slightly elevated in the FTG group. Spirochaetes and Tenericutes consistently displayed lower relative abundances in both cohorts. The genus-level comparison (right panel) revealed more pronounced inter-group differences. Specifically, the relative abundances of *Rothia* and *Neisseria* were markedly higher in the ENG group than in the FTG group. Conversely, the FTG group exhibited significantly higher relative abundances of *Filifactor* and *Streptococcus*. The distributions of numerous other genera, including *Megasphaera* and *Peptostreptococcaceae incertae sedis*, also showed varying degrees of difference between the groups. Although many genera were present at low overall abundance, their differential presence may still possess biological significance.

Following the identification of key bacterial genera exhibiting significant abundance differences between the FTG and ENG groups (**Figures 5**, **6**), we sought to gain deeper insights into the potential functional ramifications of these taxonomic shifts. Consequently, we performed comprehensive functional annotation of these differential genera, with detailed results compiled in **Supplementary Table S2**. This annotation integrates multi-dimensional information, including the potential pro- or anti-inflammatory properties of each genus, their capacity for gamma-aminobutyric acid (GABA) metabolism, notable metabolite production (particularly short-chain fatty acids, SCFAs), ecological and adaptive traits (e.g., carriage of mobile genetic elements (MGEs), biofilm formation capabilities), and potential clinical relevance (e.g., associations with periodontal pathogenesis or systemic diseases). The information presented in **Supplementary Table S1** was systematically curated from published literature and public databases (references in table footnotes). Overall, this table highlights that several genera enriched in the FTG group (e.g., *Filifactor*, *Streptococcus*, *Parvimonas*, *Tannerella*, *Treponema*) possess known pro-inflammatory potential or are associated with periodontal disease. In contrast, some genera relatively enriched in the ENG group (e.g., *Rothia*, *Neisseria*, *Cardiobacterium*) are linked to oral health or potential anti-inflammatory functions. These detailed functional annotations provide a crucial, taxon-specific foundation for subsequently exploring the functional alterations within the salivary microbiome under fatigue and their potential biological significance (see Section 2.7 and Discussion).

### 3.7 Functional prediction of the salivary bacterial community in the FTG group

**Figure 8:**
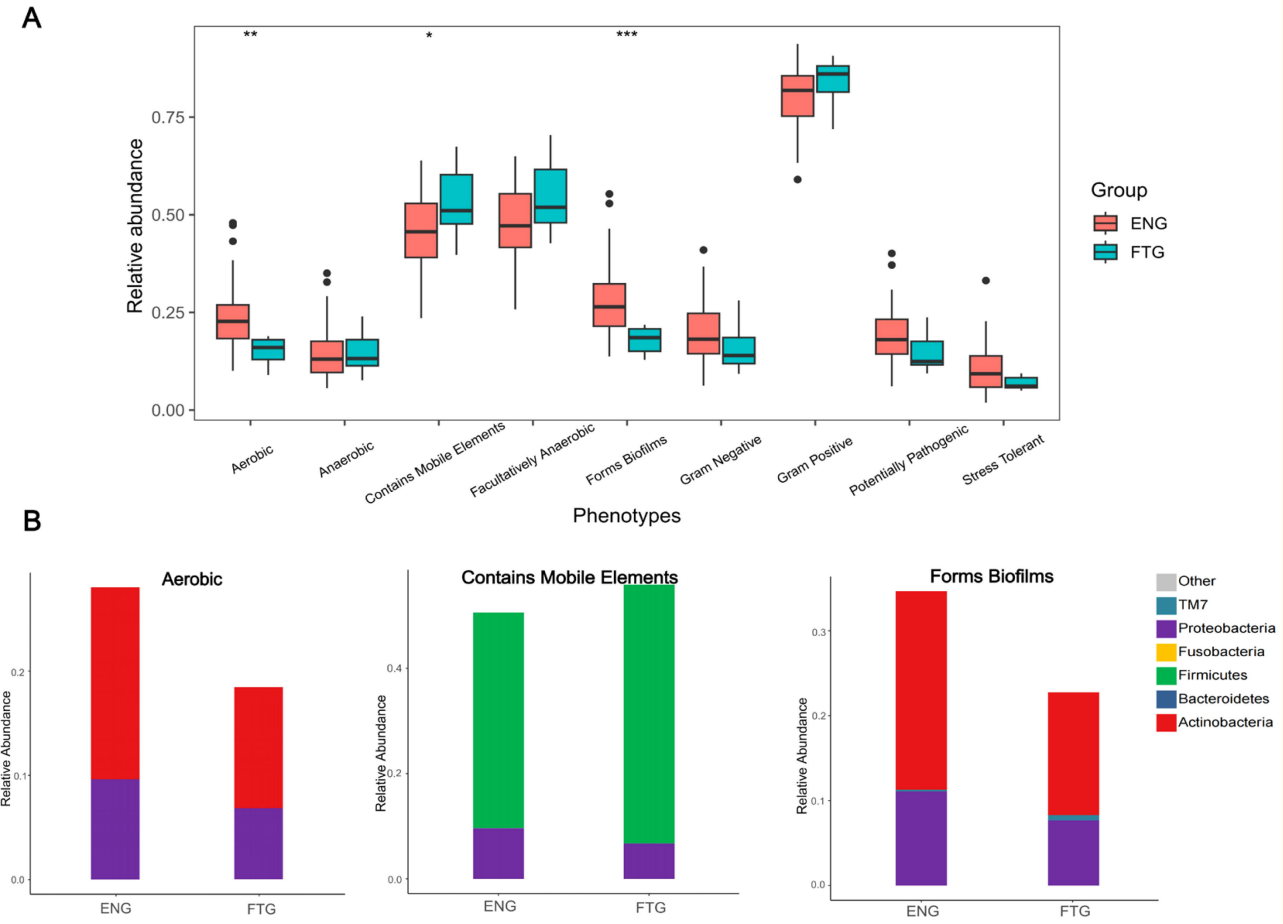
Predicted phenotype analysis of microbial communities in ENG and FTG groups. **(A)** Comparison of relative abundances for nine predicted microbial phenotypes between the two groups (box plots). Boxes represent the interquartile range (IQR), the horizontal line inside the box indicates the median, whiskers extend to data points within 1.5 times the IQR from the upper and lower quartiles, and black dots represent outliers. Group comparisons were performed using the Wilcoxon rank-sum test, with asterisks indicating statistical significance levels: *P < 0.05, **P < 0.01, ***P < 0.001. **(B)** Relative abundance composition of major taxonomic units at the phylum level within the three phenotypes showing significant inter-group differences (Aerobic, Contains Mobile Elements, Forms Biofilms) (stacked bar charts). Different colors represent different bacterial phyla as indicated in the legend.

To investigate functional phenotypic differences at the community level between the microbial communities of the ENG and FTG groups, we predicted their phenotypic profiles using BugBase analysis. The results indicated (**Figure 8A**) that, compared to the ENG group, the relative abundance of bacteria predicted to contain Mobile Genetic Elements (MGEs) was significantly higher in the FTG group (Wilcoxon rank-sum test, *P* = 0.048), suggesting a potentially higher capacity for Horizontal Gene Transfer (HGT). Conversely, the relative abundances of bacteria predicted to be Aerobic and capable of forming Biofilms were significantly lower in the FTG group (*P* = 0.006 and *P* = 0.002, respectively). No statistically significant differences were observed between the groups for other predicted phenotypes, including anaerobic, facultatively anaerobic, Gram-staining characteristics, potential pathogenicity, or stress tolerance.

Further taxonomic analysis resolved the contributions to these significantly different phenotypes (**Figure 8B**). Within the MGE-containing communities, the FTG group was primarily dominated by Firmicutes, exhibiting a significantly higher relative abundance compared to the ENG group, alongside a concomitant decrease in the relative abundance of Proteobacteria. For the Aerobic phenotype, the higher abundance in the ENG group was mainly attributed to Actinobacteria and Proteobacteria, both of which showed lower relative abundances in the FTG group. In the biofilm-forming communities, while the relative abundances of Actinobacteria and Proteobacteria were lower in the FTG group compared to the ENG group, there was a notable enrichment in the relative abundance of the TM7 phylum.

**Figure 9:**
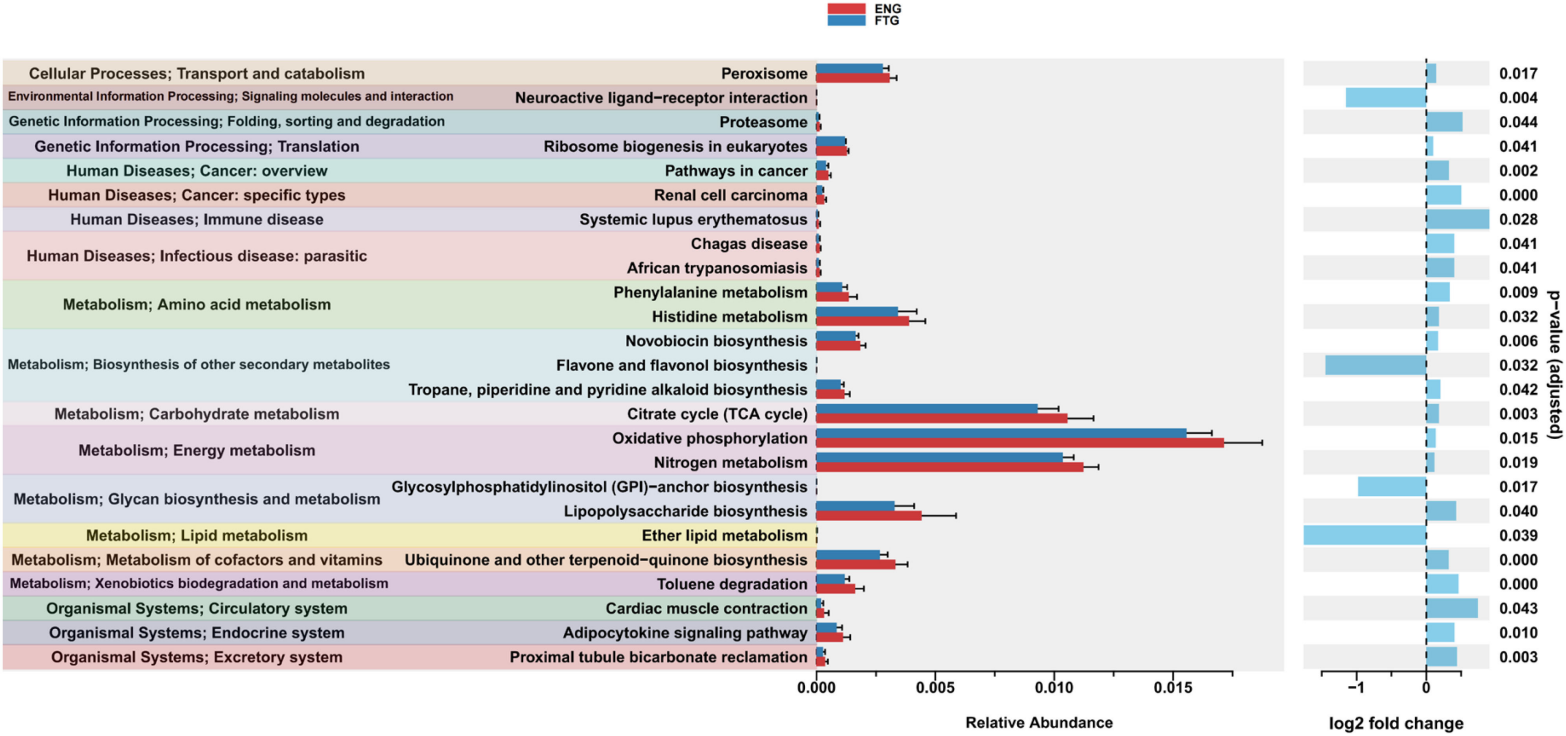
Significant differences in predicted microbial KEGG functional pathways between ENG and FTG groups. Functional profiles were predicted via ggpicrust2 and compared using LinDA method (adjusted p < 0.05). The left panel displays the mean relative abundance (± SEM) of these pathways for the ENG (red) and FTG (blue) groups, organized by KEGG Level 1 functional categories (indicated by background colors). The right panel shows the corresponding log2 fold change (calculated as FTG relative to ENG); positive values (blue bars, right) denote enrichment in the ENG group, whereas negative values (blue bars, left) denote enrichment in the FTG group. Adjusted p-values from the LinDA analysis are presented on the far right.

Furthermore, to investigate the differences in predicted functional potential of the microbial communities between the ENG and FTG groups, KEGG pathway enrichment analysis was conducted using ggpicrust2 (**Figure 9**; **Supplementary Table S5**). This predictive analysis revealed significant functional divergence between the two groups. Specifically (adjusted p < 0.05), the FTG group exhibited significant enrichment in several KEGG pathways related to Environmental Information Processing and Metabolism, including Neuroactive ligand-receptor interaction (p=0.004), Flavone and flavonol biosynthesis (p=0.032), Glycosylphosphatidylinositol (GPI)-anchor biosynthesis (p=0.017), and Ether lipid metabolism (p=0.003).

Conversely, the KEGG pathways significantly enriched in the ENG group displayed a diverse functional profile. The Metabolism category was particularly prominent, with enriched pathways broadly encompassing core energy metabolism (e.g., Oxidative phosphorylation, Citrate cycle (TCA cycle), Nitrogen metabolism), biodegradation of specific substrates (Toluene degradation), amino acid metabolism (Phenylalanine metabolism, Histidine metabolism), and various biosynthetic pathways, including those for cofactors and vitamins (Ubiquinone and other terpenoid-quinone biosynthesis), glycans (Lipopolysaccharide biosynthesis), and secondary metabolites (Novobiocin biosynthesis, Tropane, piperidine and pyridine alkaloid biosynthesis). Within Genetic Information Processing, enriched pathways included protein degradation (Proteasome) and ribosome biogenesis (Ribosome biogenesis in eukaryotes). In Cellular Processes, the Peroxisome pathway was significantly enriched. At the Organismal Systems level, enriched pathways were associated with functions analogous to host systems, such as circulatory (Cardiac muscle contraction), excretory (Proximal tubule bicarbonate reclamation), and endocrine (Adipocytokine signaling pathway) functions. Furthermore, several pathways related to Human Diseases were also significantly enriched in the ENG group, including those associated with parasitic infections (Chagas disease, African trypanosomiasis), immune diseases (Systemic lupus erythematosus), and cancer (Pathways in cancer, Renal cell carcinoma).

## 4 Discussion

Fatigue, a complex physiological and pathological state ubiquitously impacting human health, quality of life, and socioeconomic productivity, possesses underlying biological mechanisms that remain incompletely elucidated (Raizen et al., 2023; Yoon et al., 2023). In recent years, the pivotal role of the gut microbiome in the pathogenesis of fatigue, particularly Myalgic Encephalomyelitis/Chronic Fatigue Syndrome (ME/CFS), via the “microbiome-gut-brain axis” has garnered considerable attention (Guo et al., 2023; Xiong et al., 2023). However, the oral cavity, housing the second largest microbial community in the human body, represents a relatively underexplored niche concerning the association between its microbiome and fatigue states, along with potential regulatory mechanisms (Kuppuswamy, 2017; Lin et al., 2021; Raizen et al., 2023; Baker et al., 2024). This study presents the first systematic investigation into the structural and functional alterations of the salivary microbiome in healthy individuals following experimentally induced physiological fatigue (prolonged learning), aiming to uncover fatigue-associated oral dysbiosis and its potential biological significance.

Our findings clearly demonstrate significant alterations in the salivary microbiome of individuals experiencing physiological fatigue (FTG group) compared to their energetic counterparts (ENG group). Alpha diversity analysis revealed a significant decrease in the Simpson index in the FTG group (p=0.01071), suggesting reduced community evenness, potentially reflecting the overgrowth of specific dominant genera. Although other alpha diversity metrics (e.g., Shannon index) did not show statistically significant differences, beta diversity analyses based on Non-metric Multidimensional Scaling (NMDS), Analysis of Similarities (ANOSIM), and Multi-Response Permutation Procedures (MRPP) consistently confirmed statistically significant differences in the overall microbial community structure between the two groups (p<0.05). Such pronounced shifts in community structure are key indicators of microbial ecosystem imbalance (dysbiosis) (Levy et al., 2017). This observation shares similarities with reports of reduced gut microbiome diversity in some ME/CFS patients (Nagy-Szakal et al., 2017; Guo et al., 2023). However, a notable discrepancy emerged: we observed a significant increase in the relative abundance of Firmicutes in the saliva of the FTG group, while Bacteroidetes showed a non-significant downward trend. This contrasts with findings from some ME/CFS gut microbiome studies reporting elevated *Bacteroidetes* and reduced Firmicutes (Raijmakers et al., 2020). Potential explanations for this divergence include: (1) inherent differences in how microbial communities respond to physiological stress across distinct ecological niches (oral cavity vs. gut); (2) fundamental differences in microbiome signatures between physiological fatigue (focus of this study) and pathological fatigue states like ME/CFS; and (3) variations in fatigue triggers (learning stress vs. multifactorial chronic pathology) and duration, which could shape distinct microbiome alteration patterns.

At the taxonomic level, we identified several key genera exhibiting significantly different abundances between the FTG and ENG groups. The relative abundances of *Streptococcus* and *Filifactor* were significantly enriched in the FTG group. *Streptococcus*, a dominant genus in the oral cavity with diverse member functions, its overall increase could reshape the oral microecological balance, and certain species are implicated in dental caries, periodontitis, and even systemic infections (Nobbs et al., 2009; Kilian, 2018). *Filifactor*, particularly *F. alocis*, is recognized as a significant periodontal pathogen associated with periodontal tissue inflammation and bone resorption (Aruni et al., 2015). Furthermore, several anaerobic genera linked to periodontitis or opportunistic infections, such as *Treponema*, *Tannerella*, *Parvimonas*, and unclassified members of the Peptostreptococcaceae family, also showed increasing trends (though not all statistically significant) in the FTG group. Conversely, *Rothia* and *Neisseria*, often considered markers of oral health (Raijmakers et al., 2020), were significantly enriched in the ENG group. *Neisseria* participates in nitrate reduction, generating nitric oxide (NO) beneficial for cardiovascular health (Rosier et al., 2020); its reduced abundance could potentially exert adverse effects on host physiological functions. Collectively, these taxonomic shifts depict a trend wherein physiological fatigue might be accompanied by oral dysbiosis, characterized by a relative increase in potential pathogens or pro-inflammatory bacteria and a decrease in health-associated commensals. This imbalance may not only elevate the risk of oral diseases like periodontitis but could also potentially impact systemic health through pathways such as low-grade inflammation (Hajishengallis and Chavakis, 2021). Co-occurrence network analysis further suggested potential instability within the FTG microbial ecosystem: despite increased network density (more edges and nodes), the overall clustering coefficient was lower, implying weakened synergistic interactions or heightened competition among microbes, thereby disturbing homeostasis.

To explore the functional implications of these structural microbiome changes, we performed predictive analyses of biological phenotypes and functional pathways. BugBase-based phenotype prediction indicated significant phenotypic shifts in the FTG salivary microbiome compared to the ENG group. The proportion of bacteria carrying mobile genetic elements (MGEs) was significantly higher in the FTG group (p=0.048), suggesting that under fatigue-associated oral environmental stress, bacteria might adapt through more frequent horizontal gene transfer, potentially accelerating the spread of resistance or virulence genes (Partridge et al., 2018). Concurrently, the relative abundance of aerobic bacteria was significantly reduced (p=0.006), with a corresponding increase in anaerobic or facultative anaerobic bacteria. This aligns with the anaerobic nature of many pro-inflammatory genera (e.g., *Filifactor*, *Treponema*, *Tannerella*), indicating a potential shift towards a more hypoxic oral microenvironment favoring the colonization and proliferation of these potential pathogens (Lamont et al., 2018). Intriguingly, contrary to the common notion that pathogens tend to form biofilms, the predicted biofilm-forming capacity was significantly decreased in the FTG group (p=0.002). A plausible explanation is the reduced abundance of certain health-associated genera capable of forming stable protective biofilms (e.g., specific members of *Neisseria* or *Rothia*) in the FTG group (Kolenbrander et al., 2010), leading to an overall decline in the predicted biofilm formation potential.

Further functional pathway prediction using PICRUSt2 revealed deeper metabolic reprogramming features. Four key KEGG pathways were significantly upregulated (q<0.05) in the FTG group: “Neuroactive ligand-receptor interaction” (ko04080), “Glycosylphosphatidylinositol (GPI)-anchor biosynthesis” (ko00563), “Ether lipid metabolism” (ko00565), and “Flavone and flavonol biosynthesis” (ko00944). The enrichment of the “Neuroactive ligand-receptor interaction” pathway is particularly compelling, directly suggesting that oral microbes might participate in host neural signal regulation by synthesizing or metabolizing neuroactive substances (e.g., short-chain fatty acids (SCFAs), gamma-aminobutyric acid (GABA)), providing crucial molecular clues for exploring the role of the “oral-microbiome-brain axis” in fatigue development (Rea et al., 2016; Strandwitz, 2018; Bowland et al., 2022). The coordinated upregulation of GPI-anchor biosynthesis and ether lipid metabolism pathways might relate to enhanced bacterial cell surface properties (e.g., structural integrity, adhesion capacity), signal transduction, and biofilm matrix synthesis (Guo, 2023), potentially improving microbial adaptability and colonization in the oral environment. Furthermore, the activation of flavonoid biosynthesis pathways (also see ko00941 Flavonoid biosynthesis, p=0.004) suggests that oral microbes could metabolize dietary plant polyphenols (e.g., quercetin, luteolin) to produce secondary metabolites with neuro-regulatory or other bioactive properties (Braune and Blaut, 2016).

Concurrently, several core metabolic pathways exhibited significant downregulation in the FTG group, including “Phenylalanine metabolism” (ko00360, p=0.002), “Histidine metabolism” (ko00340, p=0.008), “Citrate cycle (TCA cycle)” (ko00020, p=0.01), and “Oxidative phosphorylation” (ko00190, p=0.02). This pattern of energy metabolism suppression coupled with restricted specific amino acid metabolism could promote or exacerbate fatigue through multiple mechanisms. Firstly, reduced efficiency of core energy metabolism pathways (TCA cycle and oxidative phosphorylation) directly leads to decreased cellular ATP production capacity, potentially forcing the host towards less efficient anaerobic glycolysis for energy, which might result in the accumulation of metabolic byproducts like lactate, thereby inducing or worsening peripheral fatigue (Raizen et al., 2023). Secondly, phenylalanine is a key precursor for synthesizing catecholamine neurotransmitters like dopamine and norepinephrine; downregulation of its metabolism pathway could lead to diminished dopaminergic system function, affecting motivation, reward, and attention maintenance, correlating with central fatigue (Chaudhuri and Behan, 2004; Rea et al., 2016). Similarly, histidine is the precursor for histamine; its suppressed metabolism might impact the histaminergic system and indirectly affect other neuroendocrine systems (e.g., serotonergic system) via the hypothalamic-pituitary axis, participating in fatigue regulation (Chaudhuri and Behan, 2004; Rea et al., 2016). Additionally, the “Ubiquinone and other terpenoid-quinone biosynthesis” pathway (ko00130), related to Vitamin E (a key lipid-soluble antioxidant) metabolism, was also significantly downregulated in the FTG group. Combined with reports of lower serum α-tocopherol levels in ME/CFS patients (Logan and Wong, 2001) and the fact that certain gut bacteria (like some members of *Bacteroides* and *Klebsiella*, which were less abundant in our FTG group) may participate in Vitamin E metabolism (Tarracchini et al., 2025), this suggests that fatigue states might involve perturbations in the antioxidant defense system, particularly the Vitamin E metabolic pathway. This could represent a potential mechanism linking oral microbial imbalance to fatigue and associated cognitive symptoms.

Integrating the structural and functional prediction results from this study with existing literature, we postulate that the significantly altered key microbes in the saliva of the FTG group may participate in the regulation of physiological fatigue via the “oral-microbiome-brain axis” through the following interconnected mechanisms:

### 4.1 Induction of low-grade inflammation

Oral dysbiosis, particularly the relative enrichment of potential periodontopathogens (e.g., *Treponema*, *Tannerella*, *Filifactor* elevated in our study) and the depletion of commensals with anti-inflammatory potential (e.g., *Neisseria*, *Rothia*), could lead to chronic low-grade inflammation locally in the oral cavity. These microbes, their metabolites (e.g., lipopolysaccharide, LPS), or induced pro-inflammatory cytokines (e.g., IL-1β, IL-6, TNF-α) might enter the systemic circulation through compromised oral mucosal barriers or periodontal tissues, triggering or exacerbating systemic low-grade inflammation (Liccardo et al., 2019; Hajishengallis and Chavakis, 2021). Systemic low-grade inflammation is a recognized pathophysiological feature of fatigue-related conditions like ME/CFS and can induce fatigue by affecting central nervous system functions (e.g., inducing “sickness behavior”) (Dantzer and Kelley, 2007; Morris et al., 2019).

### 4.2 Regulation of neurotransmitter metabolism and signaling

Oral microbes possess the capacity to directly synthesize or metabolize various neurotransmitters and their precursors, potentially influencing central nervous functions (Rea et al., 2016; Bowland et al., 2022).

#### 4.2.1 Dopamine signaling system

The downregulation of the phenylalanine metabolism pathway in the FTG group, combined with abundance changes in specific genera potentially involved in phenylalanine metabolism (e.g., decreased *Bacteroides*, *Klebsiella*), might collectively lead to reduced effective dopamine supply, correlating with fatigue-related symptoms like lack of motivation and anhedonia (Chaudhuri and Behan, 2004; Rea et al., 2016).

#### 4.2.2 Serotonin signaling system

Certain oral genera (e.g., *Streptococcus*, enriched in FTG) might participate in tryptophan metabolism, indirectly influencing serotonin (5-HT) availability (Jenkins et al., 2016; Rea et al., 2016). Excessive peripheral 5-HT or its metabolites could indirectly affect central 5-HT levels or function by altering blood-brain barrier permeability or directly acting on the vagus nerve; high central 5-HT levels are generally associated with increased fatigue perception (Davis and Bailey, 1997). Furthermore, the downregulation of histidine metabolism in the FTG group could affect histamine levels, thereby indirectly perturbing neuroendocrine balance, including the 5-HT system.

#### 4.2.3 GABA signaling system

Gamma-aminobutyric acid (GABA) is the primary inhibitory neurotransmitter. In the FTG group, the complex situation arising from the decreased abundance of potential GABA producers (e.g., *Megasphaera*), the reduction of GABA consumers (e.g., *Rothia*), and the enrichment of other genera potentially influencing GABA levels (e.g., *Tannerella*) might collectively lead to GABAergic system imbalance (Strandwitz, 2018). This imbalance could exacerbate fatigue by modulating hypothalamic-pituitary-adrenal (HPA) axis activity or affecting neuromuscular signaling (Cryan and Dinan, 2012).

### 4.3 Mediation via metabolites (metabolite pathway)

#### 4.3.1 Short-chain fatty acids metabolism

SCFAs are crucial microbial metabolites. Although reduced butyrate producers and lower SCFA levels are often observed in the gut of ME/CFS patients (Guo et al., 2023), our study found increased abundance of some potential butyrate-producing genera (e.g., *Filifactor*, *Tannerella*) in the saliva of the FTG group, while acetate/propionate-producing *Bacteroides* and butyrate-producing *Megasphaera* decreased. The SCFA profile changes in saliva and their local and systemic effects (if they enter circulation) might differ from those in the gut. In the context of oral inflammation, locally overproduced SCFAs (especially butyrate), if entering circulation, might induce mild neuroinflammation, oxidative stress, or participate in fatigue onset by modulating neurotransmitters (e.g., promoting peripheral 5-HT release) in the central nervous system (Dalile et al., 2019; Silva et al., 2020). However, the effects of SCFAs are concentration- and context-dependent, and their precise role (potential dual beneficial and detrimental effects) in physiological fatigue requires further investigation.

#### 4.3.2 Vitamin E metabolism

As previously mentioned, the downregulation of the ubiquinone and other terpenoid-quinone biosynthesis pathway and the decreased abundance of genera known to metabolize Vitamin E (*Bacteroides*, *Klebsiella*) in the FTG group suggest that the oral microbiome might be involved in Vitamin E metabolic balance. Considering Vitamin E’s antioxidant and neuroprotective roles and its altered levels in ME/CFS patients (Hajishengallis and Chavakis, 2021), oral microbe-mediated dysregulation of Vitamin E metabolism could be a potential mechanism linking oral dysbiosis to fatigue and related cognitive symptoms.

Notably, the combination of 15 genus-level taxa identified using the Boruta-SHAP machine learning algorithm exhibited exceptionally high predictive performance (AUC=0.948) in distinguishing between fatigued and non-fatigued states. Among these, genera such as *Rothia*, *Filifactor*, *Neisseria*, and *Streptococcus* had high importance scores (SHAP values) and were consistently identified as differential by multiple analytical methods (e.g., LEfSe, ZicoSeq, MaAsLin3, ANCOM-II, ALDEx2, PROC-GLM), reinforcing the robustness of our findings. This not only further corroborates the key roles of these genera in fatigue-associated oral microecological shifts but also highlights the potential of the salivary microbiome as a non-invasive, high-potential biomarker for assessing physiological fatigue status.

However, this study has several limitations. Firstly, the sample size of the fatigue group was relatively small (n=7), potentially limiting statistical power and the generalizability of the findings; validation in larger cohort studies is warranted. Secondly, the cross-sectional design precludes direct inference of causality from the observed associations; fatigue is dynamic, necessitating future longitudinal studies to track salivary microbiome changes throughout the onset, progression, and recovery phases of fatigue. Thirdly, while the physiological fatigue model employed (prolonged learning combined with EEG and scale assessments) offers objectivity and representativeness to some extent, its capacity to fully mimic physiological fatigue induced by diverse stressors in daily life (e.g., physical exertion, sleep deprivation, emotional stress) needs verification across varied fatigue models. Fourthly, functional predictions based on 16S rRNA sequencing (PICRUSt2) and phenotype predictions (BugBase) provide valuable functional insights, but their accuracy is constrained by the completeness of reference databases and algorithmic predictive capabilities. Future integration of multi-omics technologies, including metagenomics, metatranscriptomics, and metabolomics, will enable a more profound and precise elucidation of species/strain-level functional activities and molecular mechanisms.

In conclusion, this study provides the first systematic characterization of significant alterations in the salivary microbiome of healthy individuals following specific physiological fatigue induced by prolonged learning. These alterations encompass community structure imbalance (significant beta diversity changes, decreased evenness), shifts in key taxa abundances (e.g., enrichment of *Streptococcus*, *Filifactor*; reduction of *Rothia*, *Neisseria*), and profound reprogramming of predicted biological phenotypes (decreased biofilm formation capacity, increased proportion carrying MGEs, reduced abundance of aerobic bacteria) and functional pathways (enrichment of neuroactive ligand-receptor interaction pathway, downregulation of energy metabolism pathways, downregulation of specific amino acid metabolism pathways, changes in inflammation-related pathways). These findings strongly suggest that the oral microbial ecosystem may participate in the regulation of physiological fatigue via a potential “oral-microbiome-brain axis” through multiple pathways, including induction of low-grade inflammation, modulation of neurotransmitter metabolic networks, and production of specific metabolites (e.g., SCFAs, impacting Vitamin E metabolism). The high-accuracy predictive model based on the salivary microbiome further underscores its significant potential as a biomarker for fatigue status. Future research should focus on validating these findings through larger longitudinal cohort studies, integrating multi-omics approaches, and conducting mechanistic experiments (e.g., animal models, *in vitro* co-cultures, microbial transplantation) to elucidate specific molecular mechanisms and explore the feasibility of targeting the oral microbiome as a novel intervention strategy for ameliorating or preventing fatigue.

## Data Availability

The datasets presented in this study can be found in online repositories. The names of the repository/repositories and accession number(s) can be found below: https://data.mendeley.com/preview/tbhj7z87jz, Mendeley Data.
