# Supplementary material for: Characterization of the salivary microbiome in healthy individuals under fatigue status
Source: Front Cell Infect Microbiol. 2025 May 29;15:1506723. doi: 10.3389/fcimb.2025.1506723 (PMC12159056; doi:10.3389/fcimb.2025.1506723)
Supplement: Supplementary file 1 [file DataSheet1.docx]

**Supplement information**

**Characterization of the salivary microbiome in healthy individuals under fatigue status**


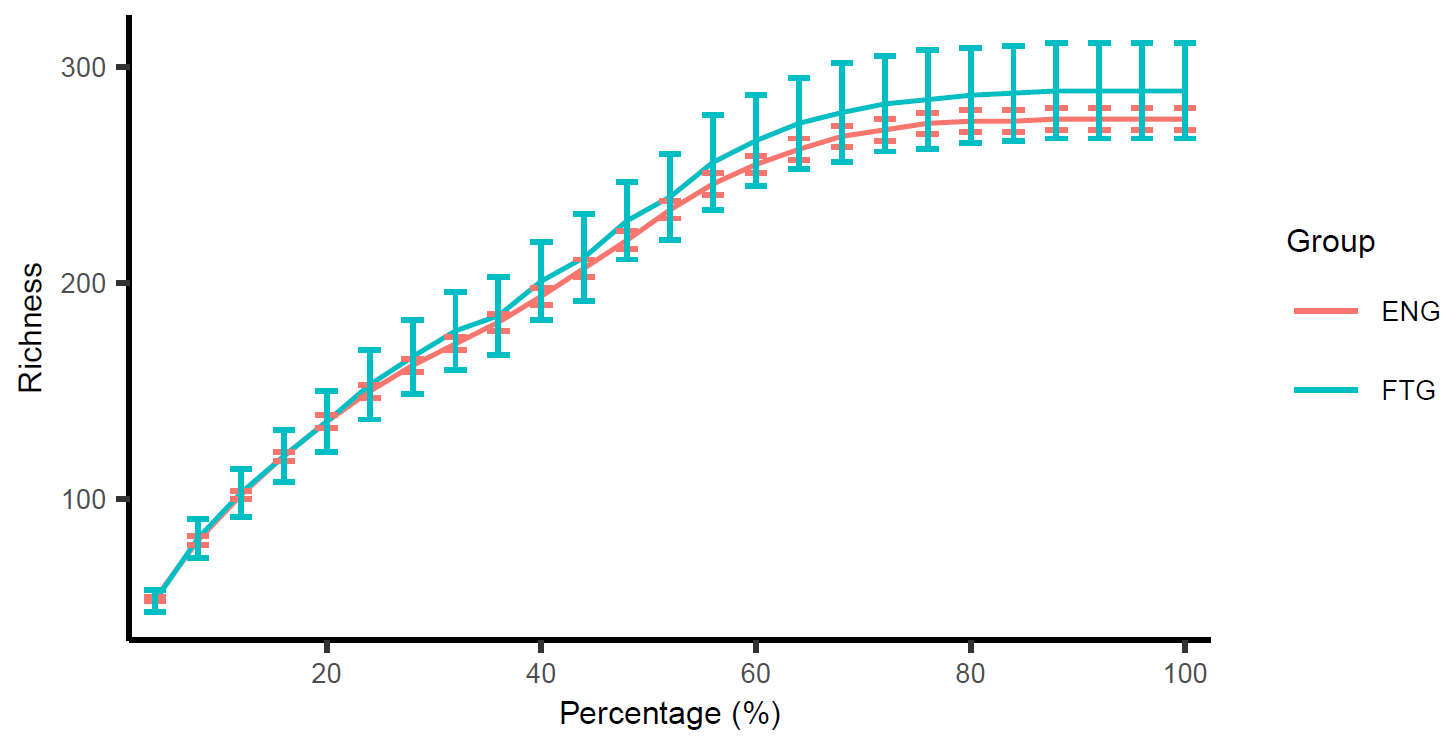


Figure S1. The distinction between species accumulation curves and rarefaction curves in the FTG and ENG groups.
