# Supplementary material for: Characterization of the salivary microbiome in healthy individuals under fatigue status
Source: Front Cell Infect Microbiol. 2025 May 29;15:1506723. doi: 10.3389/fcimb.2025.1506723 (PMC12159056; doi:10.3389/fcimb.2025.1506723)
Supplement: Supplementary file 2 [file DataSheet2.docx]

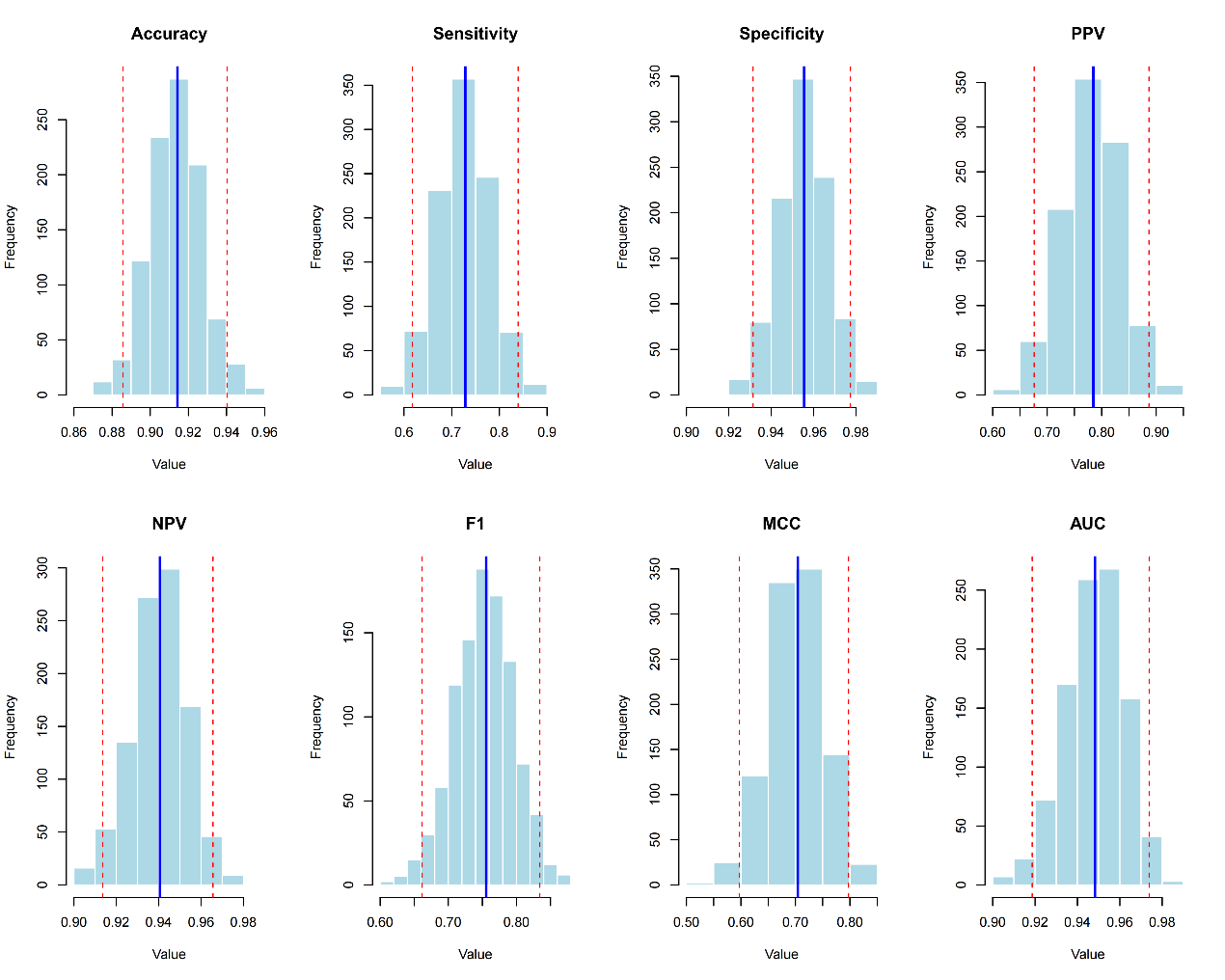


Figure S2 Distributions of Model Performance Metrics Across Resampling Iterations. This figure shows histograms of the distributions for eight key performance metrics (Accuracy, Sensitivity, Specificity, PPV, NPV, F1, MCC, AUC) obtained during model evaluation via resampling (e.g., repeated cross-validation). The solid blue line indicates the mean (or median) value for each metric, while the dashed red lines likely represent the 95% confidence interval or standard deviation bounds. This figure is used to assess the stability and reliability of the model's performance.
